# Smaller body size under warming is not due to gill-oxygen limitation in a cold-water salmonid

**DOI:** 10.1242/jeb.246477

**Published:** 2024-02-21

**Authors:** Joshua K. Lonthair, Nicholas C. Wegner, Brian S. Cheng, Nann A. Fangue, Matthew J. O'Donnell, Amy M. Regish, John D. Swenson, Estefany Argueta, Stephen D. McCormick, Benjamin H. Letcher, Lisa M. Komoroske

**Affiliations:** ^1^ Department of Environmental Conservation, University of Massachusetts Amherst, Amherst, MA 01003-9285, USA; ^2^ National Research Council under contract to Fisheries Resources Division, Southwest Fisheries Science Center, National Marine Fisheries Service, National Oceanic and Atmospheric Administration, La Jolla, CA 92037-1508, USA; ^3^ Fisheries Resources Division, Southwest Fisheries Science Center, National Marine Fisheries Service, National Oceanic and Atmospheric Administration, La Jolla 92037-1508, CA, USA; ^4^ Department of Wildlife, Fish, and Conservation Biology, University of California, Davis, Davis, CA 95616, USA; ^5^ US Geological Survey, Eastern Ecological Science Center at the S. O. Conte Research Laboratory, Turners Falls, MA 01376-1000, USA

**Keywords:** Temperature–size rule, Oxygen limitation, Ectotherm, Climate change, Brook trout

## Abstract

Declining body size in fishes and other aquatic ectotherms associated with anthropogenic climate warming has significant implications for future fisheries yields, stock assessments and aquatic ecosystem stability. One proposed mechanism seeking to explain such body-size reductions, known as the gill oxygen limitation (GOL) hypothesis, has recently been used to model future impacts of climate warming on fisheries but has not been robustly empirically tested. We used brook trout (*Salvelinus fontinalis*), a fast-growing, cold-water salmonid species of broad economic, conservation and ecological value, to examine the GOL hypothesis in a long-term experiment quantifying effects of temperature on growth, resting metabolic rate (RMR), maximum metabolic rate (MMR) and gill surface area (GSA). Despite significantly reduced growth and body size at an elevated temperature, allometric slopes of GSA were not significantly different than 1.0 and were above those for RMR and MMR at both temperature treatments (15°C and 20°C), contrary to GOL expectations. We also found that the effect of temperature on RMR was time-dependent, contradicting the prediction that heightened temperatures increase metabolic rates and reinforcing the importance of longer-term exposures (e.g. >6 months) to fully understand the influence of acclimation on temperature–metabolic rate relationships. Our results indicate that although oxygen limitation may be important in some aspects of temperature–body size relationships and constraints on metabolic supply may contribute to reduced growth in some cases, it is unlikely that GOL is a universal mechanism explaining temperature–body size relationships in aquatic ectotherms. We suggest future research focus on alternative mechanisms underlying temperature–body size relationships, and that projections of climate change impacts on fisheries yields using models based on GOL assumptions be interpreted with caution.

## Teaser

**Summary:** A long-term experiment linking individual fish growth, gill surface area and metabolism does not support the gill oxygen limitation hypothesis as a mechanism driving smaller body sizes with environmental warming.

## INTRODUCTION

Understanding the effect of temperature on the physiology and ecology of organisms is critical to predicting and mitigating the impacts of climate change. Negative temperature–body size relationships have been long observed in natural and experimental systems (Audzijonyte et al., 2019; Brett, 1979; Daufresne et al., 2009; Fry et al., 1946; Verberk et al., 2021), and this phenomenon is often broadly referred to as the temperature–size rule (TSR; but see discussion of evolution of use in Audzijonyte et al., 2019 from its origin, *sensu* Atkinson, 1994). Although the direction and strength of body size–temperature relationships can vary (Audzijonyte et al., 2020; Letcher et al., 2023; Verberk et al., 2021), negative correlations are prevalent across taxonomic groups and environments, and declining body size has been proposed as a ‘universal’ response to anthropogenic climate warming (Daufresne et al., 2009). For example, in aquatic environments where ectotherm TSR relationships are particularly strong, body sizes of many fishes have declined by an estimated average of 5–20% over the last several decades (studies summarized in Audzijonyte et al., 2019). Smaller body sizes are often linked to lower population biomass and reproductive output (Barneche et al., 2018), with significant potential implications for future fisheries yield projections, stock assessments and ecosystem stability. Some theoretical models have projected a 14–24% decrease in maximum body mass of marine fishes from 2000 to 2050 under some climate scenarios that would severely reduce catch potential in many of the world's fisheries (Cheung et al., 2011, 2013). There is also concern that body size reductions owing to warming could compound fishery-induced evolution to favor faster life histories, altering key parameters for stock and risk assessments such as per-capita population growth rate (Baudron et al., 2014; Wang et al., 2014; Waples and Audzijonyte, 2016). Yet, despite decades of research and many proposed hypotheses, we still do not have a robust understanding of the mechanism(s) underlying TSR patterns (Audzijonyte et al., 2019), without which it is extremely challenging to predict how future environmental scenarios will affect taxa within and across ecosystems.

Given the importance of forecasting ecological responses under climate warming, there has been renewed interest in examining mechanistic explanations of body size–temperature relationships (Audzijonyte et al., 2019; Verberk et al., 2021). A number of hypotheses focus on the influence of oxygen limitation on growth and aerobic scope capacities (Jutfelt et al., 2021; Verberk et al., 2021), while others are grounded in adaptive life history optimization between growth and reproduction (White et al., 2022; Wootton et al., 2022). One mechanistic hypothesis prominently discussed in the literature is the gill oxygen limitation (GOL) hypothesis (also referred to as the gill oxygen limitation theory or GOLT). The GOL hypothesis posits that in aquatic ectotherms (principally fishes) growth and other metabolically derived processes are ultimately limited by the oxygen uptake capacity of the gills (the primary oxygen uptake surface in fishes). Specifically, this hypothesis is rooted in a potential mismatch between oxygen acquisition through gill surface area (GSA, which is inherently two-dimensional) and the metabolic demands of a three-dimensional body as an organism grows (Pauly, 2021; Pauly and Cheung, 2018). This proposed mismatch between oxygen supply and demand with growth is thought to be exacerbated at warmer temperatures (under the assumption that warmer temperature increases metabolism, but see Wootton et al., 2022), thereby resulting in smaller adult body sizes.

**Fig. 1.**
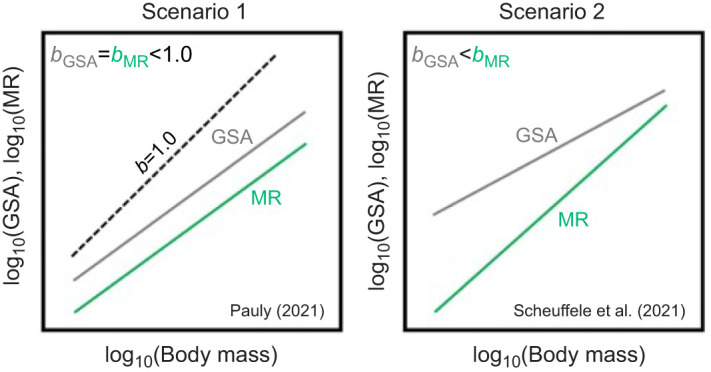
**Conceptual diagram showing hypothesized allometric slopes (*b*) for gill surface area (GSA) and metabolic rate (MR) under different proposed scenarios for gill oxygen limitation that could result in growth and body size (mass) reductions.** In scenario 1, geometric constraints on GSA (i.e. surface area to volume relationships) limit the allometric slope of GSA to less than *b*<1.0, resulting in a similarly constrained allometric slope for metabolic rate at *b*<1.0 (Pauly, 2021; Pauly and Cheung, 2018). Under scenario 2, geometric constraints result in GSA scaling less than that of MR, ultimately resulting in a mismatch between oxygen supply and demand (Scheuffele et al., 2021).

The concept of GOL has been interpreted and described in two ways (Fig. 1). First, the GOL hypothesis as described by Pauly (1981, 2021) and Pauly and Cheung (2018) is premised on the idea that organismal oxygen demand (metabolism) should scale allometrically with body mass (i.e. changes in metabolism should change proportionally with body mass as an organism grows with an allometric slope of *b*=1.0), but inherent two-dimensional geometric constraints on GSA will force both GSA (oxygen supply) and consequently metabolic rate (oxygen demand) to have allometric slopes of *b*<1.0 (Fig. 1, scenario 1). As an organism grows, Pauly (1981, 2021) and Pauly and Cheung (2018) argue this creates a potential mismatch between idealized oxygen requirements (*b*=1.0) and actual supply, and is manifested as both GSA and metabolic rate having allometric slopes of *b<*1.0. However, the physiology community largely recognizes that metabolism should not necessarily scale at *b*=1.0 to meet energetic demands (Jerde et al., 2019; Killen et al., 2010), and argues that potential GOL could alternatively be viewed as metabolic rate (oxygen demand) having a higher allometric slope than that of GSA (oxygen supply) and thus the ratio of GSA to metabolic rate decreases with body size leading to oxygen limitation with growth (Fig. 1, scenario 2; Audzijonyte et al., 2019; Scheuffele et al., 2021). However interpreted, this hypothesis has been the subject of much debate (Brander et al., 2013; Lefevre et al., 2017, 2018; Pauly, 2021; Pauly and Cheung, 2018; Roche et al., 2022), with most criticisms broadly focused around the arguments that GOL is not based on valid physiological principles or supported by existing data. For example, GOL assumes that the scaling of GSA is constrained by surface area to volume ratios, which ultimately constrain metabolic rate and growth. In contrast, critics argue that there is substantial evidence that GSA scales proportional to metabolic rate in order to meet metabolic demands (Lefevre et al., 2017, 2018; Somo et al., 2023; Prinzing et al., 2023) and that gills can be highly plastic and dynamic structures such that GSA can acclimate to changes in oxygen demand such as those induced by climate warming (Wegner and Farrell, 2023). Despite such concerns, estimates of climate impacts on fisheries have been generated by models based on GOL assumptions (Cheung et al., 2013) as well as integrated into International Union for Conservation of Nature (IUCN) climate change biological impact assessments (Cheung and Pauly, 2016). Depending on the true mechanisms of TSR and how they operate, this could mean great under- or over-estimation of climate change impacts on fisheries yields, and potentially undermine sustainable fisheries and ecosystem-based management under a warming climate (Merino et al., 2012; Ruckelshaus et al., 2013; Sumaila et al., 2011). Given that ocean and freshwater fisheries and other ecosystem resources support the livelihoods of millions of people around the globe, there is a clear need to empirically examine the support for GOL both to advance our fundamental understanding of temperature effects on body size, and to accurately forecast climate change impacts on global fisheries and aquatic ecosystems.

Several recent papers have utilized existing data in the literature to evaluate support for GOL (Bigman et al., 2021, 2023a,b; Meyer and Schill, 2021; Scheuffele et al., 2021). Although such synthesis efforts provide important insight, they are ultimately limited by constraints of datasets generated by experiments and measurements that were not designed to test this hypothesis. For example, data utilized in such synthesis studies come from a variety of different intra- and inter-specific sources, and thus generally compare non-paired growth, metabolic rate and GSA data in individuals across a range of body sizes that may not overlap (Bigman et al., 2023a,b; Lefevre et al., 2017; Pauly and Cheung, 2018; Scheuffele et al., 2021). Additionally, it is well documented that growth, metabolism and GSA can all be significantly influenced by thermal history within and across generations (Scott and Johnston, 2012; Seebacher et al., 2015; Wootton et al., 2022; Wegner and Farrell, 2023), but often data come from short-term experiments or field observations where these factors cannot be quantified or separated (Audzijonyte et al., 2019). Without careful tracking of temperature effects on growth directly paired with metabolic rate and GSA and consideration of additional context-relevant factors (e.g. where exposure temperatures are on the species' thermal performance curve or accounting for non-temperature environmental parameters), mechanistic inference from these data is limited because correlative patterns could be generated from a variety of conditions and processes. The lack of empirical datasets examining GOL and related hypotheses is understandable, as it requires logistically difficult and resource intensive experiments across temperatures and life stages with repeated measures of technically challenging and time-consuming assays. Additionally, many fish species of economic and ecological interest take months to years to reach reproductive maturity, cannot be maintained in captivity in healthy conditions for such long-term experiments, and may not be adequately represented by short-lived model species that can. Finally, such experiments require robust foundational knowledge on the thermal physiology and performance curves of target species to select temperature treatments that are appropriate to the hypotheses being tested, such as those that are expected to elicit changes in growth and metabolic demands, but not induce sublethal thermal stress responses (Verberk et al., 2021).

Here, we identified an opportunity where all of these criteria could be met to conduct a direct empirical examination of the GOL hypothesis using brook trout (*Salvelinus fontinalis*), a cold-water salmonid native to eastern North America and of high economic, ecological and conservation value (Hudy et al., 2008). The thermal physiology, reproductive biology, ecology and husbandry of this species have been extensively studied and are well characterized (Chadwick and McCormick, 2017), and the species exhibits rapid growth rates appropriate for robust trait scaling estimation (i.e. body mass increases greater than three orders of magnitude from fry to the adult life stage within 1 year). Of particular importance, the negative influence of elevated temperature on body size of this species has been established (Chadwick and McCormick, 2017), though other factors such as density dependence can play important roles in body–size relationships *in situ* (Al-Chokhachy et al., 2022). Additionally, in contrast to many model laboratory aquatic ectotherms, brook trout exhibit life-history traits representative of many real-world target fishery species (e.g. seasonal spawning; Hutchings, 1996; McCormick and Naiman, 1985) and are under threat from climate change (Trumbo et al., 2014), but can still be reared under laboratory conditions for long-term experiments (Chadwick and McCormick, 2017). Finally, because brook trout are active and streamlined swimmers, in which oxygen demands are likely high and branchial space is at a premium, this species serves as a good candidate in which to examine potential limits to gill-oxygen uptake and scaling. Leveraging this well-suited study system, we quantified the effects of temperature on the growth, metabolic rate and GSA in a long-term experiment, and then compared the allometric slopes of metabolic rate, GSA and other traits relevant to oxygen limitation to evaluate evidence for the GOL hypothesis. By conducting an explicit test of the assumptions of the GOL hypothesis, our study provides a key contribution to the growing body of knowledge on the physiological underpinnings of the TSR to enable accurate forecasting of climate warming impacts on aquatic ectotherm body size.

## MATERIALS AND METHODS

### Experimental design

Brook trout [*Salvelinus fontinalis* (Mitchill 1814)] used in these trials were spawned (five batches; each batch consisted of eggs from three to four females fertilized with milt from two males) from wild-caught adults (originally captured from a ∼1 km section of Fourmile Brook, Northfield, MA, USA) on 19 November 2020 at the US Geological Survey, Eastern Ecological Science Center, S.O. Conte Research Laboratory (Turners Falls, MA, USA). After spawning, embryos were incubated in vertical flow stacks (heath trays) with partially recirculated and temperature regulated, dechlorinated municipal water. Prior to yolk sac absorption, alevins were transferred to circular holding tanks (380 liters, 110×40 cm, diameter×height) and fed an *ad libitum* diet of dry feed (Bio Vita Starter; Bio-Oregon, Longview, WA, USA). Fish were reared at 15°C until they were 2.62±0.80 g to begin temperature growth treatments starting in July 2021. All brook trout husbandry and experimentation were completed according to approved Institutional Animal Care and Use Committee (IACUC) protocol no. 2021-07C.

Prior to growth trials, each individual was lightly anesthetized (40 mg l^−1^ Aqui-S; AQUI-S New Zealand Ltd, Lower Hutt, New Zealand) to measure initial mass, fork length (FL) and total length (TL). Passive integrated transponder (PIT) tags (8 mm HPT8; Biomark, Boise, ID, USA) were inserted into the intra-peritoneal (IP) cavity via a small incision in the abdomen. Individuals were isolated until motility was observed following anesthesia (∼5 min) to confirm recovery (99.25% recovery rate). Fish were then evenly distributed by number and size into four circular rearing tanks (380 liters, 110×40 cm, diameter×height) at their original holding temperature (15°C) for 1 week (Table S1). Following this recovery period, two tanks were kept at 15°C, while the other two were slowly (1°C day^–1^) ramped to 20°C for long-term growout over 8 months. These temperatures were selected based on previous brook trout thermal physiological research (Chadwick and McCormick, 2017) to represent an optimal growth temperature (15°C) and an elevated temperature where fish would exhibit a reduced growth rate without measurable signs of stress (20°C).

Each rearing tank was supplied with flow-through temperature-controlled freshwater (supplied via collection from a well) at a rate of ∼120 l h^−1^ to promote water mixing and directional flow. Photoperiod matched the seasonal day length via a mixture of natural light and artificial light via timers. Water temperature and mortality were checked daily, and ammonia, nitrate and dissolved oxygen were measured weekly. Temperature loggers (HOBO Water Temperature Pro v2 Data Logger; Onset Computer Corporation, Bourne, MA, USA) were placed in each tank to record water temperature at 10 min intervals and downloaded weekly to confirm temporal stability of thermal conditions (mean±s.d.; 15°C replicate 1=15.2±0.2°C; 15°C replicate 2=15.2±0.2°C; 20°C replicate 1=20.2±0.3°C; 20°C replicate 2=20.1±0.3°C). Animals were fed 2–4% body mass daily based on animal size; as animals grew, the percent body mass fed decreased. Seventy percent of total food was fed via automatic feeders, whereas the remaining 30% was fed by hand to confirm fish were fed to satiation each day in each tank.

Metabolic rate data, including resting metabolic rate (RMR), maximum metabolic rate (MMR), aerobic scope and *Q*_10_ (the relative change of metabolic rate over a 10°C increase in temperature; Clarke and Fraser, 2004), were collected at the 2 week, 3 month and 6 month post-temperature-ramping time points for a random subset of individuals (Table S2) using standard respirometry techniques detailed below. The same individuals that underwent metabolic rate measurements were lethally sampled at the conclusion of respirometry trials to collect GSA measurements, which provided a matching set of gill measurements at the 2 week, 3 month and 6 month time points.

### Growth and condition metrics

All individuals within a replicate were measured monthly (a total of seven measurements across the experiment) for mass (g), FL (mm) and TL (mm) to quantify growth metrics and body condition factor. Upon completion of all experiments in March 2022, a final set of growth metrics was collected for all remaining fish across temperature trials to determine growth over the entire 8-month period. Specific growth rate (SGR) was calculated using the equation:
(1)

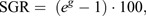
where
(2)

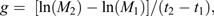
and where *M*_1_ and *M*_2_ are the average live body masses (g) at times *t*_1_ and *t*_2_, respectively (Jobling, 1983). Relative body condition factor (*K*) was calculated using the equation:
(3)

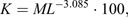
where *M* is live body mass (g) and *L* is fork length (cm) of each fish (Ricker, 1975). The 3.085 coefficient was selected after plotting fork length versus mass for all fish in study and using a regression analysis to interpret the exponent (Letcher and Terrick, 2001).

Cortisol was also measured in a subset of individuals as a proxy for sublethal temperature stress (Wendelaar Bonga, 1997). Eight individuals per replicate from each treatment were randomly lethally sampled at 2 weeks, 3 months and 6 months for blood collection via caudal vessels using a heparinized syringe. Plasma cortisol concentrations were measured in all samples collected within 5 min of tank disturbance using a validated direct competitive enzyme immunoassay as described by Carey and McCormick (1998). The standard curve ranged from 1 to 320 ng ml^−1^. Sensitivity as defined by the standard curve was determined to be 0.3 ng ml^−1^. The average inter-assay variation was 6.22% and intra-assay variation was 3.23%.

### Respirometry

#### Experimental set-up

Intermittent-flow respirometry (Clark et al., 2013; Steffensen, 1989; Svendsen et al., 2016) was used to obtain brook trout oxygen consumption rates (*Ṁ*_O_2__) as proxies for aerobic metabolic rate to quantify the effects of temperature and body size on metabolism. Respirometers were composed of an acrylic tube holding chamber with a bolted acrylic lid and recirculating plumbing of PVC connectors and flexible plastic tubing (Tygon, Saint-Gobain, Malvern, PA, USA) specific to the size range of brook trout at different time points (Table S2). The 2 week and 3 month respirometry measurements were completed using prefabricated respirometry chambers (Loligo Systems, Viborg, Denmark; total system volumes of 0.657 and 0.727 liters, respectively), whereas the 6 month measurements were made using custom-built chambers (total system volume of 4.012 liters).

A fiber optic oxygen sensor probe (Presens, Regensburg, Germany) was placed within the recirculating loop of each respirometer, each of which was connected to one of two 4-port oxygen meters (Witrox 4, Loligo Systems), with data acquisition software (AutoResp, Loligo Systems) allowing for the collection of dissolved oxygen measurements from up to eight individual respirometers at once. Dissolved oxygen measurements were collected within each respirometer at a frequency of 1 Hz. Each respirometer was mixed and flushed using water pumps (LEDGLE, China or Eheim, Deizisau, Germany, depending on respirometer size). Respirometers were submerged in a temperature-regulated water bath (258 liters, 245×55.5×19 cm, length×width×height) to ensure that the temperature was kept constant at 15 or 20°C depending on the individual's acclimation temperature (Table S2). Additional details about respirometry design and trials can be found in Table S5.

#### Maximum metabolic rate

Following a 24 h fasting period, brook trout were individually subjected to a chase protocol followed by measurement of *Ṁ*_O_2__ to estimate MMR. In brief, a fish was individually transferred from its rearing tank to a chase tank (48 liters, 87×20 cm, diameter×depth) and aggressively chased by hand until exhaustion following protocols as described in previous studies (Durhack et al., 2021; Mochnacz et al., 2017). Immediately following the chase, the fish was placed in the respirometry chamber and the lid was quickly bolted in place to minimize the time (<1 min) to the first measurements of oxygen consumption. After closing the chamber, the fish was allowed to consume oxygen until the dissolved oxygen was 80–90% air saturation. MMR was then estimated as the highest rate of oxygen uptake over a 1-min time window using a rolling regression in R (https://www.r-project.org/; version 4.2.1; Prinzing et al., 2021).

#### Resting metabolic rate

Following MMR measurements, each fish was allowed to recover from exercise within its respirometry chamber for 24 h before beginning RMR measurements (Durhack et al., 2021), which were then recorded over the subsequent 24 h to allow for a full diel cycle. Using an automated program (AutoResp, Loligo Systems), intermittent-flow respirometry trials were divided into three phases or cycles – flush, wait and measure – with durations calibrated to the fish and chamber sizes for each of the three sampling periods (2 week, 3 months and 6 months; see details in Table S3). Flush cycles turned on an auxiliary pump and allowed for new water to enter the respirometer through check valves to return oxygen levels to ∼100% air saturation. Following each flush cycle, the auxiliary flush pump turned off, resealing the chamber for measurement of oxygen depletion by the fish. *Ṁ*_O_2__ was calculated for each fish from the resulting oxygen depletion traces following removal of the non-linear data associated with the lag of water circulation from closure of the chamber (‘wait’ period, Table S3). By convention, an *R*^2^ threshold of 0.9 was used for each RMR oxygen depletion trace following removal of the wait period, with the mean±s.d. *R*^2^ exceeding this threshold for all trials (2 week trials: 0.98±0.05; 3 month trials: 0.97±0.03; and 6 month trials: 0.91±0.08). If an individual oxygen depletion trace fell below the 0.9 *R*^2^ threshold, that trace was removed and was not included in further analysis (Killen et al., 2021). For each individual fish, RMR was estimated as the average of the lowest 25% of all RMR *Ṁ*_O_2__ measurements, which eliminated higher *Ṁ*_O_2__ measures that are not reflective of resting metabolism (Killen et al., 2021).

#### Hypoxia tolerance

Following RMR measurements, *P*_crit_ (the dissolved oxygen level at which fish aerobic metabolism can no longer be maintained) was estimated for each fish via a closed circuit draw down of dissolved oxygen saturation (Ultsch and Regan, 2019). The respirometer flush pump was shut off while the recirculation pump was left operational in order to maintain water mixing within the chamber. Dissolved oxygen saturation was measured at a frequency of 1 Hz, and dissolved oxygen consumption was allowed to continue until the fish experienced a loss of equilibrium. Upon loss of equilibrium, the flush pump was immediately activated, and the fish was allowed to recover. *P*_crit_ was then estimated using the ‘respirometry’ package (https://cran.r-project.org/web/packages/respirometry/index.html) and the LLO method (Reemeyer and Rees, 2019), as the *P*_O_2_ _at which *Ṁ*_O_2__ falls below RMR projected as a line from normoxia to anoxia.

#### Background respiration

Prior to MMR measurements and following *P*_crit_ measurements, background respiration was measured within the respirometer (without the fish) for 1 h. Background respiration was modeled over time linearly as a percentage of RMR and subtracted from *Ṁ*_O_2__ measurements.

### Gill surface area dimensions

Following the respirometry trial, each individual was euthanized with an overdose of the anesthetic tricaine mesylate (MS-222) buffered with sodium bicarbonate (100 mg l^−1^ MS-222; pH 7.4), patted dry and measured for mass and length. The head including all gill arches was removed and fixed in buffered 10% formalin. GSA for each fish was estimated as:
(4)

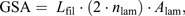
where *L*_fil_ is the total length (mm) of all gill filaments on both sides of the head, *n*_lam_ is the lamellar frequency (i.e. the mean number of lamellae per unit length on one side of a filament, multiplied by two to account for lamellae on both sides of the filament) and *A*_lam_ is the mean bilateral surface area of a lamellae (Wegner, 2011; Wegner et al., 2010).

To make GSA measurements, all four gill arches were removed from the right side of the head. All filaments on all eight hemibranchs were counted and evenly divided into five to seven bins per hemibranch, with approximately 12 filaments per bin. A magnified photo (Amscope SM-1 Series, Irvine, CA, USA) was taken of the median filament in each bin, which was assumed to be representative of all filaments in that bin. The length of this median filament was traced and measured using imaging software (ImageJ, National Institutes of Health, Bethesda, MD, USA, Java 1.8.0_172) following methods detailed in Wegner et al. (2010) and Wegner (2011). The total length of all filaments (*L*_fil_) on all hemibranchs on the right side of the head was calculated by multiplying the length of the median filament in each bin by the total number of filaments in that bin, then summing the length of all filaments in all bins. This length was doubled to account for the length of filaments on the left side of the head that were not measured.

Following filament measurements, lamellar frequency and lamellar surface area measurements were made on all median filaments from all eight hemibranchs on the first dissected brook trout individual. These measurements showed that the posterior hemibranch on the second gill arch was most representative of the gills as a whole, and thus for subsequent brook trout individuals, lamellar frequency and mean lamellar surface area measurements were based solely on this hemibranch (Wegner, 2011; Wegner et al., 2010). To make lamellar frequency and lamellar surface measurements, the median filament from each bin was viewed under the dissection scope. A magnified photo was taken of the tip and base half of the median filament using imaging software (ImageJ) for estimation of lamellar frequency (number of lamellae per mm). A cross-section was then made at the tip, middle and base locations on the median filament, which was then turned on its side to take a magnified photograph of the extended lamellae on both sides of the filament for a lamellar surface area measurement at the tip, middle and base of each filament. Lamellar surface area (mm^2^) was estimated by tracing the outline of the lamella on one side of the filament at all locations, then doubling it to represent the bilateral surface area of the lamella.

Lamellar frequency (*n*_lam_, mm^−1^) was estimated by averaging lamellar frequency measurements taken at each location (base and tip) of each individual filament, multiplying this mean by the total length of all filaments in that bin to give the total number of lamellae per bin, summing the total number of lamellae in all bins, then dividing this by the total length of all filaments on the hemibranch. Average lamellar surface area (*A*_lam_, mm^2^) was estimated by taking the mean of lamellar surface area measurements taken at the three locations (tip, middle and bottom) on each filament, multiplying this mean by the total number of lamellae in that bin to give a total lamellar area per bin, summing the total lamellar area for all bins, then dividing by the total number of lamellae on the hemibranch.

### Statistical analysis

The impacts of temperature and time on mass (g), SGR and condition factor (*K*) were analyzed with separate generalized linear mixed models (GLMMs), using categorical fixed effects of time (days in experiment) and temperature (°C) as predictors. For random effects, fish identity was initially nested within tank to account for repeated measures over time and tank effects. However, the random tank effect was subsequently removed because estimated variances were zero or near zero and models did not substantively change with this modification. For growth and condition factor, the data were non-normal, positive and continuous, so a Gamma error distribution was used with log link function. SGR data were continuous and freely ranging about zero, so a Gaussian error distribution was used with identity link function. Growth data were characterized by heteroscedasticity because SGR declined and was less variable with age. In contrast, condition factor exhibited more variation with age because animals were selected for similar size to start the experiment. To account for this, the dispersion (variance) parameter for growth and condition factor models was allowed to vary with time (Brooks et al., 2017).

To quantify allometric slopes of GSA, MMR and RMR, power-law regressions were modeled using log_10_-transformed body size data as a predictor with log_10_-transformed GSA, MMR (absolute) and RMR (absolute) data as individual response metrics. This allowed for quantification of the scaling or allometric slope (*b*) and *y*-intercept (*a*) across body mass (*x*) for any metric (*y*) in the form of the power equation *y*=*ax^b^* or log form log_10_*y*=*a*+*b*log_10_*x*. For these models, temperature was initially included as an additional predictor to determine whether there were temperature effects on scaling, but for the individual allometric slopes, parameter estimates are reported based on data from each temperature treatment alone. The same analytical approach was used for quantifying allometric slopes for the components of GSA (total filament length, lamellar frequency and lamellar area). The difference in the allometric slope of GSA to RMR and MMR (*b*_S_; Scheuffele et al., 2021) was calculated at each temperature as:
(5)

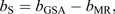
in which *b*_S_≥0 indicates a divergence of the allometric slopes of GSA and MR (inconsistent with scenario 2), whereas *b*_S_≤0 indicates a convergence of the allometric slopes of GSA and MR (consistent with scenario 2).

To allow for direct evaluation of how metabolism changed across temperature and time independent of changes to fish body size, MMR and RMR were mass corrected (scaled; MR_mc_) to a geometric mean body mass (*M*_geomean_) of 24.0 g using the equation:
(6)

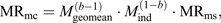
where *M*_ind_ is individual mass and MR_ms_ is mass-specific MR, using the temperature-specific allometric slopes of MMR and RMR. Mass-corrected estimates of MMR and RMR were used to calculate factorial aerobic scope (FAS) (mass-corrected MMR/mass-corrected RMR) across temperatures (15°C and 20°C) and time points (2 weeks, 3 months and 6 months). Likewise, the *Q*_10_ for mass-corrected RMR and mass-corrected MMR was also calculated for the 15°C and 20°C temperature acclimations at the 2 week, 3 month and 6 month time points using the equation:
(7)

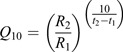
where *R*_1_ and *R*_2_ are the metabolic rate (MMR or RMR) at temperatures *T*_1_ and *T*_2_. The impacts of temperature and time were then assessed on mass-corrected metabolic rates, as well as cortisol and *P*_crit_ using linear models with the same categorical fixed effects as described above (time and temperature) as predictors but without random effects because the measures were lethal and not repeated on individuals. For gill metric, metabolic rate, cortisol and *P*_crit_ models, a Gaussian error distribution was used and Type II ANOVA tables (if no interactions present) or Type III ANOVA tables (if interactions were present) were calculated. For *post hoc* contrasts, marginal means and Tukey's adjustment for multiple comparisons were estimated. Models were evaluated by examining diagnostics including a posterior predictive check, linearity and heteroscedasticity tools using ‘performance’ and simulated residuals using ‘DHARMa’ (Ludecke et al., 2021; https://CRAN.R-project.org/package=DHARMa). In the case of cortisol, data were log_10_ transformed to improve homoscedasticity. All statistical analysis was completed in R (version 4.2.1) and using the packages ‘tidyverse’ (Wickham et al., 2019), ‘glmmTMB’ (Brooks et al., 2017) and ‘emmeans’ (https://CRAN.R-project.org/package=emmeans). A summary of all statistical analyses is provided in Table S4A. The data and code for all statistical analysis are available on Github (see Data Availability statement).

## RESULTS

### Temperature effects on growth and condition metrics

**Fig. 2.**
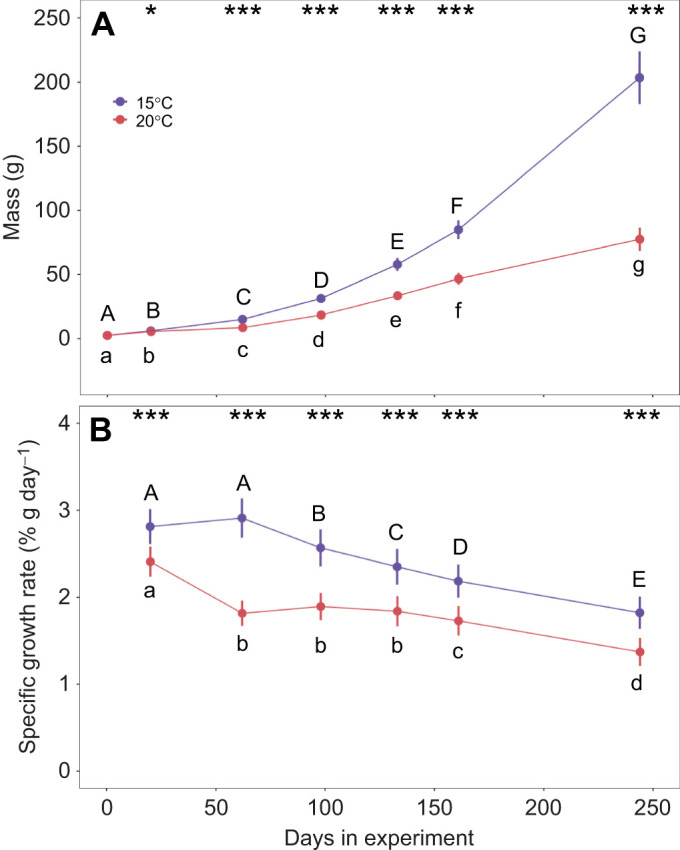
**Effect of temperature on body mass and specific growth rate in the brook trout, *Salvelinus fontinalis*, over 8 months of growout.** (A) Body mass (g; mean±s.e.m.); (B) specific growth rate (% g day^−1^; mean±s.e.m.). Asterisks refer to differences between temperature treatments within a time step (*P<0.05, ***P<0.001). Letters above and below means denote *post hoc* testing results across time but within a temperature (15°C, uppercase; 20°C, lowercase) at ɑ<0.05. For body mass, data were log_10_ transformed prior to analysis but are shown in raw form (means±s.e.m.) here for interpretation. *N*-values for each treatment and time point are available in Table S1.

Increased temperature negatively affected brook trout body size and growth rates (Fig. 2A,B). Specifically, fish held at 20°C had significantly lower body masses (Fig. 2A) associated with lower SGRs (Fig. 2B) than fish held at 15°C starting at the first time point (20 days), and these differences continued to compound over time. By the end of the experiment, fish attained over a 77-fold increase in body mass in the 15°C treatment, as opposed to only a 30-fold increase in body mass in the 20°C treatment (initial mean±s.e.m. mass: 2.62±0.04 g; final mass at 15°C: 203.43±6.64 g and final mass at 20°C: 77.50±3.93 g); final body mass for both treatments exceeded the size of first maturation for the species (∼50 g; B.L. personal observation). Temperature had a negative effect on body mass (Wald χ^2^=95.503, *P*<0.001; Table S4B) whereas time had a positive effect on body mass (Wald χ^2^=52384.189, *P*<0.001; Table S4B), and there was evidence for an interaction of these two factors (Wald χ^2^=942.824, *P*<0.001; Table S4B). Likewise, temperature (Wald χ^2^=205.85, *P*<0.001; Table S4B) and time (Wald χ^2^=2209.36, *P* <0.001; Table S4B) both had negative effects on SGR, and there was also evidence for an interactive effect (Wald χ^2^=366.46, *P*<0.001; Table S4B). *Post hoc* analyses revealed that fish held at 20°C displayed significantly lower body masses (Fig. 2A) and SGRs (Fig. 2B) relative to those at 15°C at every time point measured across the entire duration of the experiment.

There were small but significant changes in relative condition factor (*K*), where fish acclimated at 20°C had a slightly higher condition factor than fish acclimated at 15°C (Fig. S1A). Statistical analysis showed an effect of temperature (Wald χ^2^=32.709, *P*<0.001; Table S4B) and time on condition factor (Wald χ^2^=103.537, *P*<0.001; Table S4B), and there was evidence for an interaction (Wald χ^2^=135.044, *P*<0.001; Table S4B). *Post hoc* tests revealed no evidence for changing relative condition factor over time within the 15°C treatment but variable changes over time within the 20°C treatment (Fig. S1A).

For plasma cortisol levels, modeling revealed some evidence for an effect of temperature (*F*_1,80_=4.275, *P*=0.041), time (*F*_2,80_=12.999, *P*<0.001) and an interaction (*F*_2,80_=5.154, *P*=0.011). *Post hoc* testing indicated that plasma cortisol levels were greater in the 20°C treatment relative to the 15°C treatment at 20 and 190 days, but not 98 days (Fig. S1B). Within both temperature treatments, cortisol generally increased over time (Fig. S1B).

### Allometric slopes of metabolic rate and gill surface area

**Fig. 3.**
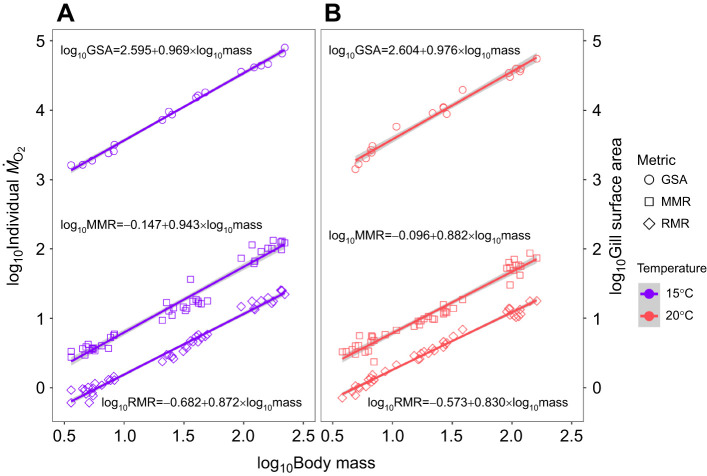
**Relationships of gill surface area (GSA; mm^2^), resting metabolic rate (RMR; mg** **O_2_** **h^–1^) and maximum metabolic rate (MMR; mg** **O_2_** **h^–1^) with body mass (g) at 15°C and 20°C in the brook trout, *Salvelinus fontinalis*.** Scaling equations are provided for each trait (*y*) in the form of log_10_*y*=*a*+*b*log_10_*M*, where *a* is the intercept at 1 g, *b* is allometric slope and *M* is mass (g). 95% confidence intervals are represented by gray shading. *N*-values for each treatment are available in Table S2.

**Fig. 4.**
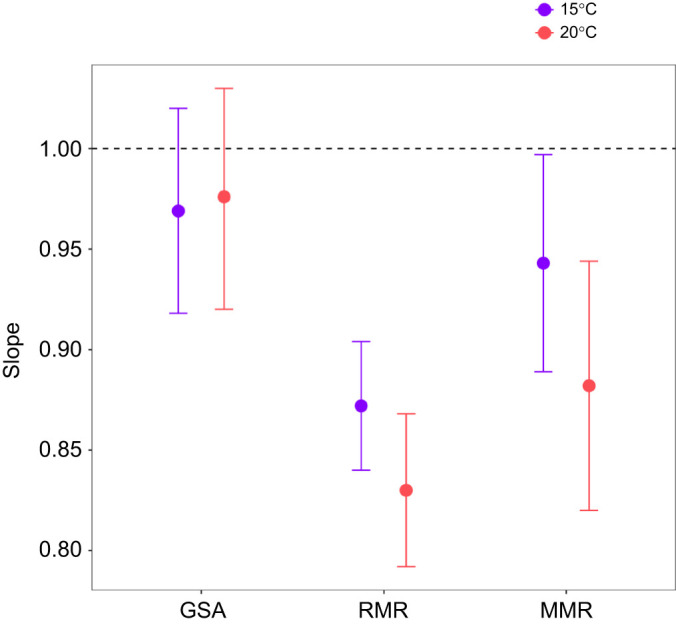
**Brook trout allometric slope (*b*) estimates and associated 95% confidence intervals for gill surface area (GSA), resting metabolic rate (RMR) and maximum metabolic rate (MMR) at 15°C and 20°C.** *N*-values for each treatment are available in Table S2.

**Fig. 5.**
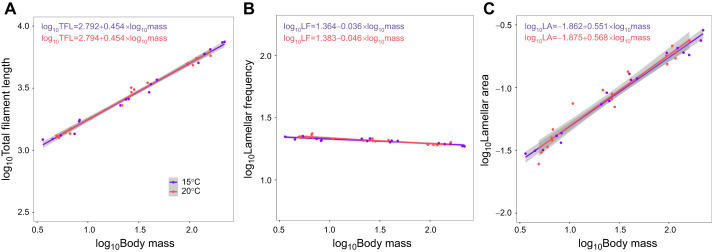
**Scaling relationships of gill surface area dimensions in the brook trout, *Salvelinus fontinalis*.** (A) Total filament length (mm), (B) lamellar frequency (count mm^–1^) and (C) bilateral lamellar surface area (mm^2^) at 15°C and 20°C. 95% confidence intervals are represented by gray shading.

The allometric slope of GSA (i.e. change in GSA with body mass) was greater than 0.95 at both temperatures (15°C: *b*_GSA_=0.969; 20°C: *b*_GSA_=0.976), with upper confidence intervals both encompassing *b*=1.0 (Figs 3, 4), in conflict with scenario 1 (Fig. 1). The allometric slopes and associated confidence intervals for RMR (15°C: *b*_RMR_=0.872, *b*_S_=0.097; 20°C: *b*_RMR_=0.830, *b*_S_=0.146) and MMR (15°C: *b*_MMR_= 0.943, *b*_S_=0.026; 20°C: *b*_MMR_=0.882, *b*_S_=0.094) either overlapped or were lower than corresponding GSA values (*b*_S_>0; Figs 3, 4), in conflict with expectations of GOL under scenario 2 (Fig. 1). Analysis further revealed that body mass was an important driver of GSA (*F*_1,32_=2719.395, *P*<0.001), whereas temperature (*F*_1,32_=0.801, *P*=0.377) and the body mass and temperature interaction were not (*F*_1,32_=0.036, *P*=0.850). Similarly for MMR, body mass was a significant predictor (*F*_1,82_=2012.073, *P*<0.001), whereas temperature (*F*_1,82_=1.974, *P*=0.164) and the body mass by temperature interaction had no effect (*F*_1,82_=2.162, *P*<0.145). In contrast, for RMR, body mass was a strong significant predictor (*F*_1,81_=4876.825, *P*<0.001), and there was also evidence of a temperature effect (*F*_1,81_=13.982, *P*<0.001) but no indication of an interaction (*F*_1,81_=2.884, *P*=0.093). Examination of the allometric slopes of the gill metrics that compose GSA revealed that total filament length (*L*_fil_) and lamellar area (*A*_lam_) comprised the majority of the GSA relationship with body size (*L*_fil_ mean slopes=0.454 for both temperatures; *A*_lam_ mean slopes=0.551 and 0.568 for 15°C and 20°C, respectively; Fig. 5), whereas the contribution of lamellar frequency (*n*_lam_) was minimal (*n*_lam_ mean slopes= –0.036 and −0.046 for 15°C and 20°C, respectively).

### Temperature effects on metabolic rate and hypoxia tolerance

**Fig. 6.**
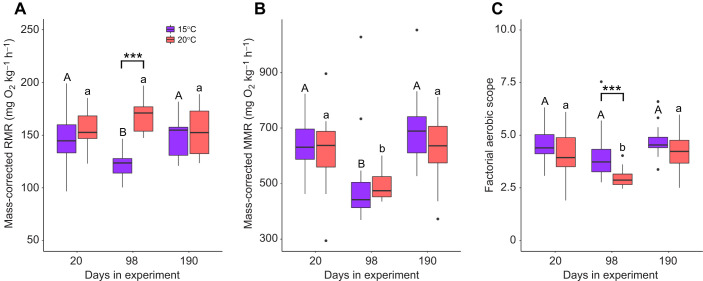
**Effects of thermal acclimation at 15°C and 20°C over time on brook trout, *Salvelinus fontinalis*.** (A) Resting metabolic rate (RMR), (B) maximum metabolic rate (MMR) and (C) factorial aerobic scope (FAS). To eliminate the effect of body size and allow for more direct comparison, all metabolic data were mass corrected to a common mean body mass of 24.0 g across temperatures and time points using temperature-treatment specific scaling exponents (Fig. 3, see statistical analysis methods). Data are displayed as means±s.e.m. Days in experiment represents the start date of respirometry trials that were completed over a 2 week period. Asterisks refer to differences between temperature treatments within a time point (***P<0.001). Within a temperature treatment, results of *post hoc* testing are referenced with letters denoting grouping (15°C, uppercase; 20°C, lowercase) at ɑ<0.05. *N*-values for each treatment and time point are available in Table S2.

Analysis of mass-corrected RMR revealed no effect of temperature (*F*_1,79_=0.002, *P*=0.963), but there was evidence of a time effect (*F*_2,79_=8.741, *P*<0.001) and a temperature×time interaction (*F*_2,79_=9.980, *P*<0.001). *Post hoc* tests showed that mass-corrected metabolic rate (RMR) was elevated in fish held at 20°C relative to those at 15°C at 3 months, but not at 2 weeks or 6 months (Fig. 6A). Within temperature treatments, no significant differences in mass-corrected RMR were detected at 20°C over time; however, in the 15°C treatment, mass-corrected RMR was significantly lower at 3 months (Fig. 6A). The mass-corrected (MMR) model revealed evidence for an effect of time (*F*_2,80_=13.904, *P*<0.001) but not temperature (*F*_1,80_=2.810, *P*=0.098) or an interaction (*F*_2,80_=0.485, *P*=0.618). *Post hoc* testing showed no evidence for temperature effects within each time point, but for both temperature treatments, mass-corrected MMR was significantly lower at 3 months (Fig. 6B). Finally, the FAS model revealed effects of temperature (*F*_2,77_=10.561, *P*=0.002) and time (*F*_2,77_=7.403, *P*=0.001) but no evidence for an interaction (*F*_2,77_=1.146, *P*=0.323). *Post hoc* tests showed that FAS was reduced in the 20°C treatment, but only at 3 months, not at 2 weeks or 6 months. Within temperature treatments, FAS was invariant over time within the 15°C treatment but was significantly lower at 3 months in the 20°C treatment (Fig. 6C).

**Fig. 7.**
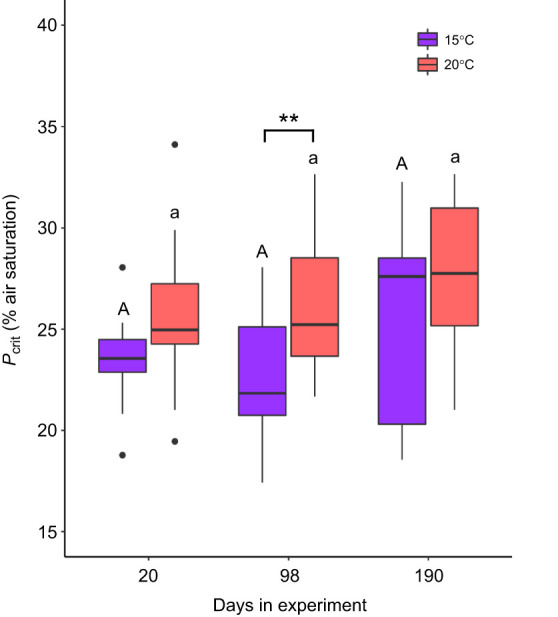
**Effect of long-term temperature acclimation on brook trout, *Salvelinus fontinalis*, critical oxygen level (*P*_crit_, dissolved oxygen level at which aerobic metabolism can no longer be maintained) as a measure of hypoxia tolerance.** Asterisks refer to differences between temperature treatments within a time point (**P<0.01). Within a temperature treatment, results of *post hoc* testing are referenced with letters denoting grouping (15°C, uppercase; 20°C, lowercase) at ɑ<0.05. *N*-values for each treatment are available in Table S2.

*P*_crit_ was significantly elevated at 20°C relative to 15°C at 3 months, but not the 2 week or 6 month time points (Fig. 7). Modeling revealed a temperature effect on *P*_crit_ (*F*_1,71_=10.339, *P*=0.002), but no evidence for an effect of time (*F*_2,71_=2.769, *P*=0.069) or an interaction (*F*_2,71_=0.415, *P*=0.661). The *Q*_10_ analysis for mass-corrected RMR revealed a similar pattern, where a *Q*_10_ value of close to 1.0 was observed at 2 weeks and 6 months (*Q*_10_=1.08 and 0.99) but was higher at 3 months (*Q*_10_=1.74). In contrast, for mass-corrected MMR, the *Q*_10_ values were close to 1.0 across all time points (2 weeks=0.84, 3 months=0.99 and 6 months=0.76).

## DISCUSSION

Mechanistic understanding of correlative patterns observed in nature is essential for accurate prediction of species' responses to environmental change. Our study is the first to empirically measure and integrate the three key variables involved in GOL (growth, metabolic rate and GSA) over a long enough timescale to evaluate support for the hypothesis directly. Our results demonstrate that although brook trout have reduced growth with increased temperature, the allometric slopes for metabolic rate metrics and GSA were clearly not aligned with either interpretation (scenario 1 or 2; Fig. 1) of the GOL hypothesis. Additionally, the effect of temperature on brook trout metabolic rate was time dependent, with brook trout at 15 and 20°C showing no significant difference in metabolic rate after 2 weeks or 6 months of temperature acclimation. This is in conflict with the underlying assumption of the GOL hypothesis (as well as some other hypotheses) that metabolic demands increase with temperature regardless of long-term acclimation processes, and is concordant with other recent work showing similar metabolic temperature insensitivity across generations (Wootton et al., 2022). This emphasizes the need for longer-term experiments to fully evaluate biological responses to temperature. Thus, although it is possible that GOL could occur in some species under certain environmental contexts, our work strongly indicates it is unlikely to be a universal mechanism driving the widespread TSR patterns observed in aquatic ectotherms. Given the importance of understanding the drivers of body size–temperature relationships for thermal biology, fisheries ecology and future food security, it is imperative that efforts be directed toward understanding alternative TSR mechanisms.

One challenge to examining evidence for GOL is that it has been interpreted to predict different possible relationships (i.e. scenario 1 versus scenario 2). However, both scenarios of the GOL hypothesis are based on the supposition that the scaling of GSA, a two-dimensional surface, cannot scale at the same rate as a three-dimensional body. Indeed, geometric isometry (proportional growth or expansion of the body in all dimensions) would predict surface areas should scale with an allometric slope of 2/3 (area/volume). Although it is clear that GSA in most fishes scales higher than the predicted slope of 2/3, the GOL hypothesis as interpreted in scenario 1 predicts it cannot scale as high as 1.0 and thus the scaling of metabolic rate is also limited to less than 1.0 (scenario 1), or because GSA cannot scale as high as metabolic rate, the ratio of GSA to metabolic rate will decrease with growth (scenario 2; Audzijonyte et al., 2019; Scheuffele et al., 2021). Our results show that brook trout GSA scales close to 1.0 and has a higher mean allometric slope than both RMR and MMR (*b*_S_>0; Scheuffele et al., 2021), contradicting both scenarios. If there is no evidence for GSA-based limitation on MMR or RMR in the highly active brook trout (in which oxygen demands are relatively high and branchial space may be more limited owing to cranial streamlining), it seems unlikely that GOL would apply to other animals, particularly those with relatively lower oxygen requirements and GSAs.

The high allometric slope of GSA shows that the gills of brook trout, like in other fishes, do not grow isometrically, and can be further understood by breaking down the scaling of individual gill components. Specifically, geometric isometry would predict that filament length should have an allometric slope of 0.33 (length/volume), lamellar frequency an allometric slope of −0.33 (length^−1^/volume) and lamellar surface an allometric slope of 0.67 (surface area/volume), which, when summed (0.33–0.33+0.67), result in a total of GSA allometric slope of *b*=0.67 (Wegner, 2011; Wegner and Farrell, 2023). However, in most fishes, the lamellar frequency allometric slope commonly scales higher than geometric isometry would predict. This is because the thickness of the lamellae and spacing in between adjacent lamellae do not greatly increase with body mass (Lefevre et al., 2018; Wegner and Farrell, 2023). This allows the allometric slope of lamellar frequency to approach 0 (instead of −0.33), allowing GSA to have an allometric slope approaching 1.0 in some species (Palzenberger and Pohla, 1992; Wegner et al., 2010). Here, we found that the brook trout achieves a high GSA allometric slope by minimizing changes to lamellar frequency (*b*=–0.036 to −0.045 depending on the temperature; Fig. 5), resulting a GSA allometric slope close to 1.0 under both temperature regimes.

Thus, despite clear decreases in growth and some increases in metabolic rate with temperature, we did not find detectable differences in GSA associated with temperature (Fig. 3). Although phenotypic plasticity of GSA in response to both temperature and hypoxia have been documented across a broad expanse of fishes (Chapman, 2007; Nilsson, 2011; Sollid and Nilsson, 2006; Wegner and Farrell, 2023), lack of such differences in the brook trout may suggest the gills in this species are already sufficiently buffered to deal with such changes. This seems likely for a species that exhibits large differences in energy expenditure and is naturally subjected to variable temperature and oxygen content both seasonally and when moving between habitats (e.g. flowing streams versus more stagnant pools). Indeed, in the closely related rainbow trout, *Oncorhynchus mykiss*, only approximately 58% of the gill lamellae are perfused with blood while at rest (Booth, 1978). The recruitment of additional perfused lamellae during exercise and elevated temperature seems an appropriate mechanism for increasing functional gill area to meet variable metabolic demand. It is possible that greater differences in temperature treatments would have resulted in detectable differences in GSA or diffusion distances, though this was outside of the scope of the present study.

Our finding that brook trout had lower growth rates at 20°C compared with 15°C throughout the experiment (Fig. 2B) is consistent with previous experiments (Chadwick and McCormick, 2017) and field estimates (Childress and Letcher, 2017) indicating that some mechanism(s) limit growth in this species at higher temperatures. Metabolic demands are typically expected to be heightened at higher temperatures, and several TSR hypotheses, including but not limited to GOL, are grounded in this assumption (Audzijonyte et al., 2019). We observed higher metabolic rates at 20°C compared with 15°C, but only at the 3 month time point. Broadly, our metabolic rate measures were similar in magnitude to previous studies (Durhack et al., 2021), suggesting that higher temperatures can increase metabolic demands. However, the lack of significant differences at the 2 week and 6 month time points emphasizes that these temperature–metabolic relationships are complex, perhaps reflecting time or age-specific acclimation effects. Although we cannot tease apart these possible drivers in the present study, the presence of a temperature–time interaction effect on metabolism reinforces both the need to examine these factors in greater depth to fully evaluate different TSR mechanisms (Wootton et al., 2022), and the fact that our reductions in growth rate are not explained by elevated metabolism alone.

We selected treatment temperatures of 15°C and 20°C in this study expected to elicit changes in growth and metabolic demands, but still be under ‘benign’ conditions (Verberk et al., 2021) such that thermal stress would not impair growth. Chadwick and McCormick (2017) found that brook trout growth rates became negative at 23.5°C, and plasma cortisol levels (as a metric for sublethal stress) were elevated above 24°C after 24 days of exposure, but were not different among 16°C, 18°C or 20°C treatments. We found generally similar results, with initial low cortisol levels that increased with size in both temperatures but remained within normal expected ranges for this species (Fig. S1), but we did observe a significant effect of temperature on cortisol in the 2 week and 6 month time points. Cortisol is known to increase metabolic rate and decrease growth rate in salmonids and most other teleosts (Mommsen et al., 1999; Vargas-Chacoff et al., 2021), but the moderate temperature-related increases in plasma cortisol we observed did not seem to appreciably affect RMR (which did not differ with temperature when cortisol was elevated) or growth rate (which was consistently 10–20% lower at 20°C throughout the study independent of cortisol level). Additionally, the slightly higher body condition factor of fish acclimated to 20°C compared with 15°C (Fig. S1A) suggests that long-term exposure did not result in a reduction in overall physical condition of brook trout at the elevated temperature treatment. Collectively, these results indicate that the strong reductions in growth and body size we observed at the higher temperature were not primarily driven by sublethal stress.

As GOL does not appear responsible for widespread observations of smaller body sizes with warmer temperatures, we must focus our attention on alternative mechanisms for accurate projections of climate warming impacts. Audzijonyte et al. (2019) and Verberk et al. (2021) recently reviewed evidence for a variety of potential TSR mechanisms, both highlighting the need for additional studies and recognition that body size may be influenced by different, and possibly multiple, mechanisms depending on temporal scales as well as species physiology and ecology. This likely explains recent empirical support for life history and physiological-based mechanisms; in other words, these hypotheses are not necessarily mutually exclusive because they focus on certain aspects of temperature–size relationships in different contexts. For example, in contrast to GOL, which focuses on constraints of oxygen uptake at the gills, it has also been proposed that oxygen delivery to tissues could be a proximate limiting factor with increased metabolic demand under warming (Clark et al., 2008). This is consistent with our finding of a significant decrease in the allometric slope of RMR at warmer temperatures (and a similar trending, but not significant, decrease in MMR), without a concomitant change in GSA scaling (Fig. 4). Indeed, exercise studies on other salmonids have shown clear limits in cardiovascular performance at higher temperatures which appear strongly correlated with upper thermal limits in some species (Clark et al., 2008; Eliason et al., 2011; Gilbert et al., 2019; Steinhausen et al., 2008). Additionally, reduced food consumption above *T*_opt_ has been observed in numerous species including brook trout (Chadwick and McCormick, 2017), which has been hypothesized to result in reduced growth rates from the need to ‘protect’ aerobic scope (Jutfelt et al., 2021). In contrast, Wootton et al. (2022) found compelling evidence for life-history based drivers (e.g. trade-offs between growth and reproduction). Furthermore, reductions in interspecific competitive ability of brook trout at elevated temperatures has been previously identified, which has possible implications for foraging efficiency and growth (Hitt, et al., 2017). Verberk et al. (2021) as well as White et al. (2022) emphasize the intricate connections between metabolism, growth and reproduction that may link potential proximate physiological mechanisms to fitness, ultimately creating selective pressures that shape TSR patterns over evolutionary timescales (e.g. ghost of oxygen-limitation past; Verberk et al., 2021) and life-history optimization (White et al., 2022).

Finally, it is important to recognize that challenges to explain body size–temperature relationships have arisen in part from inconsistent application of the TSR concept in the literature over time. Although Atkinson (1994) originally proposed TSR specifically to describe how ectotherms in warmer conditions grow faster during the juvenile phase followed by slower growth rates to ultimately attain smaller adult body sizes (i.e. a form of developmental plasticity within a population), TSR was later applied more broadly to experimental and field observations across populations and even species, where different selective pressures and microevolutionary mechanisms likely play key roles in shaping body size responses to temperature (e.g. the influence of season length producing local adaptation among populations, *sensu* Conover et al., 2009; see more detailed discussion of TSR history in Audzijonyte et al., 2019). Additionally, although some hypotheses assume processes are occurring on the rising portion of a thermal performance curve (TPC), others focus on the falling portion (Jutfelt et al., 2021) or do not specify (e.g. GOL). These seemingly nuanced differences may have important implications for how adult body size–temperature patterns arise; unfortunately, most species for which correlative TSR patterns have been observed in nature do not have available TPC data, so these factors cannot currently be assessed. Thus, advancing our understanding of body size–temperature relationships and predicting biological responses to climate warming likely needs to include well-designed experimental tests of individual hypotheses, as well as integrative work determining their relative importance and connections under different temporal, ecological and evolutionary contexts.

Identifying the drivers of shrinking body sizes under climate warming is clearly a pressing need for advancing our fundamental understanding of temperature–body size relationships as well as forecasting impacts on ecosystems and fisheries resources. Given the lack of support for GOL being a likely mechanism for these patterns, our work highlights the urgent need for future studies examining alternative hypotheses using a diversity of ectotherm species, ecological contexts and longer-term temporal scales to robustly understand these complex patterns in nature.

## Supplementary Material

10.1242/jexbio.246477_sup1 Supplementary information
