# Supplementary material for: Smaller body size under warming is not due to gill-oxygen limitation in a cold-water salmonid
Source: J Exp Biol. 2024 Feb 21;227(4):jeb246477. doi: 10.1242/jeb.246477 (PMC11093110; doi:10.1242/jeb.246477)
Supplement: Supplementary information [file jexbio-227-246477-s1.pdf]

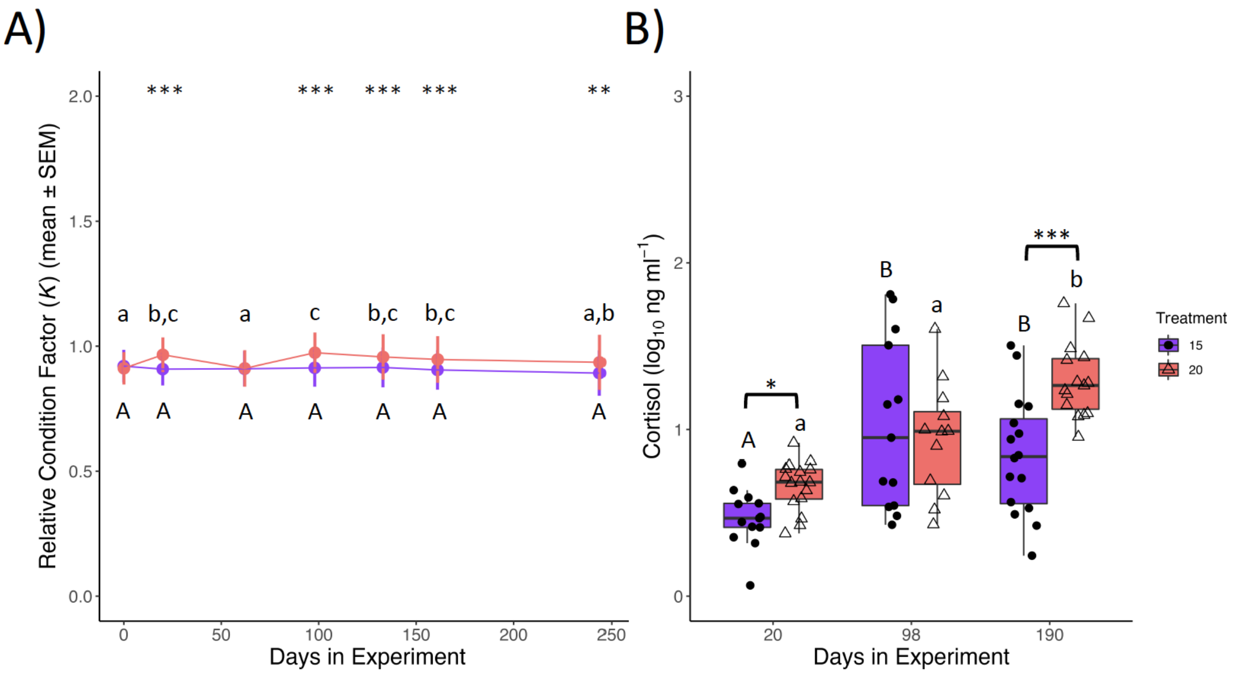

Fig. S1. Effects of thermal acclimation at 15°C and 20°C on brook trout *Salvelinus fontinalis* (A) relative condition factor (*K*) and (B) cortisol (ng/mL) as metrics of sublethal stress and health. Data in (A) is displayed as mean±sem. Asterisks refer to differences between temperature treatments within a time point (* = P < 0.05, ** P < 0.01, *** P < 0.001). Within a temperature treatment, results of post-hoc testing are referenced with letters denoting grouping (15°C = uppercase, 20°C = lowercase) at α < 0.05. *N* for each treatment are available in Table S1 (A) and Table S2 (B).

Journal of Experimental Biology • Supplementary information

Table S1. Sample sizes (N) of brook trout, *Salvelinus fontinalis*, based on treatment (15°C and 20°C) and trial replicate (1 and 2) throughout the 8 month grow out study. Time points match those used for growth analysis.

Time Point	15°C (Replicate 1)	15°C (Replicate 2)	20°C (Replicate 1)	20°C (Replicate 2)
0 days	100	99	99	99
20 days	97	98	97	98
62 days	83	82	81	76
98 days	74	74	75	70
133 days	66	66	58	53
161 days	66	66	52	51
244 days	48	49	36	36

Table S2. Mean temperature (± SD), sample size, and body mass for respirometry trials, GSA, and plasma cortisol metrics of brook trout, *Salvelinus fontinalis*, following acclimation to different temperatures (15°C and 20°C) for 2 weeks, 3 months, or 6 months.

Treatment	Temperature (°C)	Metric	N *(per replicate)*			Mass		
			2 weeks	*3 months*	*6 months*	*2 weeks*	*3 months*	*6 months*
15°C	14.7 ± 0.3	Respirometry	8	8	6	5.7 ± 1.5	34.6 ± 9.2	162.6 ± 42.5
15°C	14.7 ± 0.3	Plasma Cortisol	8	8	8	6.0 ± 2.0	35.8 ±14.2	84.4 ± 38.3
15°C	14.7 ± 0.3	GSA	3	3	3	6.2 ± 2.0	33.2 ±11.2	158.1 ± 49.4
20°C	19.4 ± 0.4	Respirometry	8	8	6	6.0 ± 1.3	22.9 ± 7.2	112.8 ± 21.7
20°C	19.4 ± 0.4	Plasma Cortisol	8	8	8	5.2± 1.8	18.8 ± 9.3	70.1 ± 22.5
20°C	19.4 ± 0.4	GSA	3	3	3	6.0 ± 0.8	25.5 ± 9.1	115.8 ± 24.4

Table S3. Respirometer chamber volume and intermittent flow cycle times used to make metabolic measurements of different sized brook trout, *Salvelinus fontinalis*, throughout the 8 month growout.

Time Point	Chamber Volume (L)	Flush Cycle (s)	Wait Cycle (s)	Measure Cycle (s)
2 weeks	0.657	180	180	600
3 months	0.727	300	30	120
6 months	4.012	340	100	160

Journal of Experimental Biology • Supplementary information

Table S4A and S4B.

Available for download at
https://journals.biologists.com/jeb/article-lookup/doi/10.1242/jeb.246477#supplementary-data

Table S5. Checklist of 53 essential criteria for the reporting of methods for aquatic intermittent-flow respirometry.

Number	Criterion and Category	Response	Value (where required)	Units
	**EQUIPMENT, MATERIALS, AND SETUP**			
**1**	Body mass of animals at time of respirometry	Variable – Please see Figure 2 for full scope.		g
**2**	Volume of empty respirometers with tubing	Variable:<br>0.657 L (2 week)<br>0.726 L (3 month)<br>4.104 L (6 month)		L
**3**	How chamber mixing was achieved	**2 Week:**<br>Recirculation Pump (LEDGLE)<br>Flush Pump (LEDGLE)<br>**3 Month:**<br>Recirculation Pump (LEDGLE)<br>Flush Pump (LEDGLE)<br>**6 Month:**<br>Recirculation Pump (LEDGLE)<br>Flush Pump (Eheim)	LEDGLE - 180 L/h<br>Eheim - 300 L/h	
**4**	Ratio of net respirometer volume (plus any associated tubing in mixing circuit) to animal body mass	2 week: Range 183:1 to 75:1<br>3 month: Range 67:1 to 15:1<br>6 month: Range 45:1 to 18:1		
**5**	Material of tubing used in any mixing circuit	Vinyl		
**6**	Volume of tubing in any mixing circuit	Was measured in combination with respirometers not independently.		
**7**	Confirm volume of tubing in any mixing circuit was included in calculations of oxygen uptake	Yes		
**8**	Material of respirometer (e.g. glass, acrylic, etc.)	Acrylic		

**9**	Type of oxygen probe and data recording	Presens Fibre Optic Dipping Probes		
**10**	Sampling frequency of water dissolved oxygen	1 Hz		
**11**	Describe placement of oxygen probe (in mixing circuit or directly in chamber)	Mixing Circuit		
**12**	Flow rate during flushing and recirculation, or confirm that chamber returned to normoxia during flushing	Chambers returned to normoxia during flushing		
**13**	Timing of flush/closed cycles	Variable:<br>2 Week – Flush = 180 s<br>2 Week – Closed = 600 s<br>3 Month – Flush = 300 s<br>3 Month – Closed = 120 s<br>6 Month – Flush = 340 s<br>6 Month – Closed = 160 s		
**14**	Wait (delay) time excluded from closed measurement cycles	2 Week – Wait = 180 s<br>3 Month – Wait = 30 s<br>6 Month – Wait = 100 s		
**15**	Frequency and method of probe calibration (for both 0 and 100% calibrations)	Probes were calibrated using over-aerated water for 100% calibration and 1 g of sodium sulfite per 100 mL of solution for 0% calibration every time before respirometry trial.		
**16**	State whether software temperature compensation was used during recording of water oxygen concentration	No – compensated in post		
	**MEASUREMENT CONDITIONS**			
**17**	Temperature during respirometry	Variable	15°C or 20°C	
**18**	How temperature was controlled	Water batch with heat pump / chillers		
**19**	Photoperiod during respirometry	Day/Night cycle of natural environment		
**20**	If (and how) ambient water bath was cleaned and aerated during measurement of oxygen uptake (e.g. filtration, periodic or continuous water changes)	Water bath was cleaned and periodic water change occurred before every respirometry run		
**21**	Total volume of ambient water bath and any associated reservoirs	~561 L		
**22**	Minimum water oxygen dissolved oxygen reached during closed phases	80%		

Journal of Experimental Biology • Supplementary information

**23**	State whether chambers were visually shielded from external disturbance	No		
**24**	How many animals were measured during a given respirometry trial (i.e. how many animals were in the same water bath)	6 – 8 animals		
**25**	If multiple animals were measured simultaneously, state whether they were able to see each other during measurements	Animals were able to see each other		
**26**	Duration of animal fasting before placement in respirometer	48 hours		
**27**	Duration of all trials combined (number of days to measure all animals in the study)	12 days		
**28**	Acclimation time to the laboratory (or time since capture for field studies) before respirometry measurements	Animals were spawned in laboratory setting on November 19th, 2020 and experiments were initiated in July 2021		
	**BACKGROUND RESPIRATION**			
**29**	State whether background microbial respiration was measured and accounted for, and if so, method used (e.g. parallel measures with empty respirometry chamber, measurements before and after for all chambers while empty, both)	Microbial respiration was measured and accounted for by measuring before and after for all chamber while empty.		
**30**	State if background respiration was measured at beginning and/or end, state how many slopes and for what duration	Background respiration was measured at beginning and end, with one slope over a 1 hour timer period per chamber.		
**31**	State how changes in background respiration were modelled over time (e.g. linear, exponential, parallel measures)	We completed a linear regression across time.		
**32**	Level of background respiration (e.g. as a percentage of SMR)	mean of 2.63% across all trials		
**33**	Method and frequency of system cleaning (e.g. system bleached between each trial, UV lamp)	Systems were bleached (10% solution) between each trial		
	**STANDARD OR ROUTINE METABOLIC RATE**			
**34**	Acclimation time after transfer to chamber, or alternatively, time to reach beginning of metabolic rate measurements after introduction to chamber	24 hours post MMR measurement		
**35**	Time period, within a trial, over which oxygen uptake was measured (e.g. number of hours)	24 hours		

Journal of Experimental Biology • Supplementary information

**36**	Value taken as SMR/RMR (e.g. quantile, mean of lowest 10 percent, mean of all values)	Mean of lowest 25%		
**37**	Total number of slopes measured and used to derive metabolic rate (e.g. how much data were used to calculate quantiles)			
**38**	Whether any time periods were removed from calculations of SMR/RMR (e.g. data during acclimation, periods of high activity [e.g. daytime])	24 hour acclimation period		
**39**	r^2 threshold for slopes used for SMR/RMR (or mean)	$r^2 = 0.9$		
**40**	Proportion of data removed due to being outliers below r-squared threshold	mean of 7.9% across all trials		
	**MAXIMUM METABOLIC RATE**			
**41**	When MMR was measured in relation to SMR (i.e. before or after)	Before		
**42**	Method used (e.g. critical swimming speed respirometry, swim to exhaustion in swim tunnel, or chase to exhaustion)	Chase to exhaustion method		
**43**	Value taken as MMR (e.g. the highest rate of oxygen uptake value after transfer, average of highest values)	Highest rate of oxygen uptake value after transfer for a give 1 minute period.		
**44**	If MMR measured post-exhaustion, length of activity challenge or chase (e.g. 2 min, until exhaustion, etc.)	Until exhaustion		
**45**	If MMR measured post-exhaustion, state whether further air-exposure was added after exercise	No further air exposure was added		
**46**	If MMR measured post-exhaustion, time until transfer to chamber after exhaustion or time to start of oxygen uptake recording	< 1 minute		
**47**	Duration of slopes used to calculate MMR (e.g. 1 min, 5 min, etc.)	1 minute		
**48**	Slope estimation method for MMR (e.g. rolling regression, sequential discrete time frames)	Rolling regression		
**49**	How absolute aerobic scope and/or factorial aerobic scope is calculated (i.e. using raw SMR and MMR, allometrically mass-adjusted SMR and MMR, or allometrically mass-adjusting aerobic scope itself)	Allometrically mass-adjusted SMR and RMR		
	**DATA HANDLING AND STATISTICS**			
**50**	Sample size	Variable<br>2 week = 16 per temperature		

Journal of Experimental Biology • Supplementary information

		treatment<br>3 month = 16 per temperature treatment<br>6 month = 12 per temperature treatment		
**51**	How oxygen uptake rates were calculated (software or script, equation, units, etc.)	R script – available via Github		
**52**	Confirm that volume (mass) of animal was subtracted from respirometer volume when calculating oxygen uptake rates	Yes		
**53**	State whether analyses accounted for variation in body mass and describe any allometric mass-corrections or adjustments	Scaling coefficients were calculated independently for MMR and RMR for both temperature treatments. And data was allometrically mass-corrected before completing two-way ANOVA analysis.		
